# The complete mitochondrial genome of *Chlaenius bimaculatus* Dejean, 1826 (Coleoptera: Carabidea) and its phylogenetic analyses

**DOI:** 10.1080/23802359.2024.2397993

**Published:** 2024-09-04

**Authors:** Jun Li, Bowei Zhou, Ying Chen, Wenlong Jiao, Qilian Zheng, Yinghua Tong

**Affiliations:** a College of Forestry, Fujian Agriculture and Forestry University, Fuzhou, China; b Key Laboratory of Integrated Pest Management in Ecological Forests, Fujian Province University, Fujian Agriculture and Forestry University, Fuzhou, China; c College of Plant Protection, Fujian Agriculture and Forestry University, Fuzhou, China; d Forestry Bureau of Lianjiang County, Fuzhou, China

**Keywords:** *Chlaenius bimaculatus*, Carabidea, mitochondrial genome, phylogenetic analysis

## Abstract

*Chlaenius bimaculatus* Dejean, 1826 (Coleoptera: Carabidea) is a predator of several lepidopteran pests, including *Spodoptera frugiperda*, *S. litura* and *Helcystogramma triannulella*. However, there has been little research into using *C. bimaculatus* to control crop pests. In this study, we sequenced the complete mitochondrial genome of *C. bimaculatus*. The results showed that the entire mitochondrial genome was 16,419 bp and contained 24% GC. 13 protein-coding, 22 transfer RNA, and two ribosomal RNA genes were identified. *C. bimaculatus* shares the same genetic arrangement and composition as other Coleoptera insects. In addition, phylogenetic analysis revealed that *C. bimaculatus* is closely related to *Diplocheila zeelandica*.

## 1. Introduction

**Figure 1.**
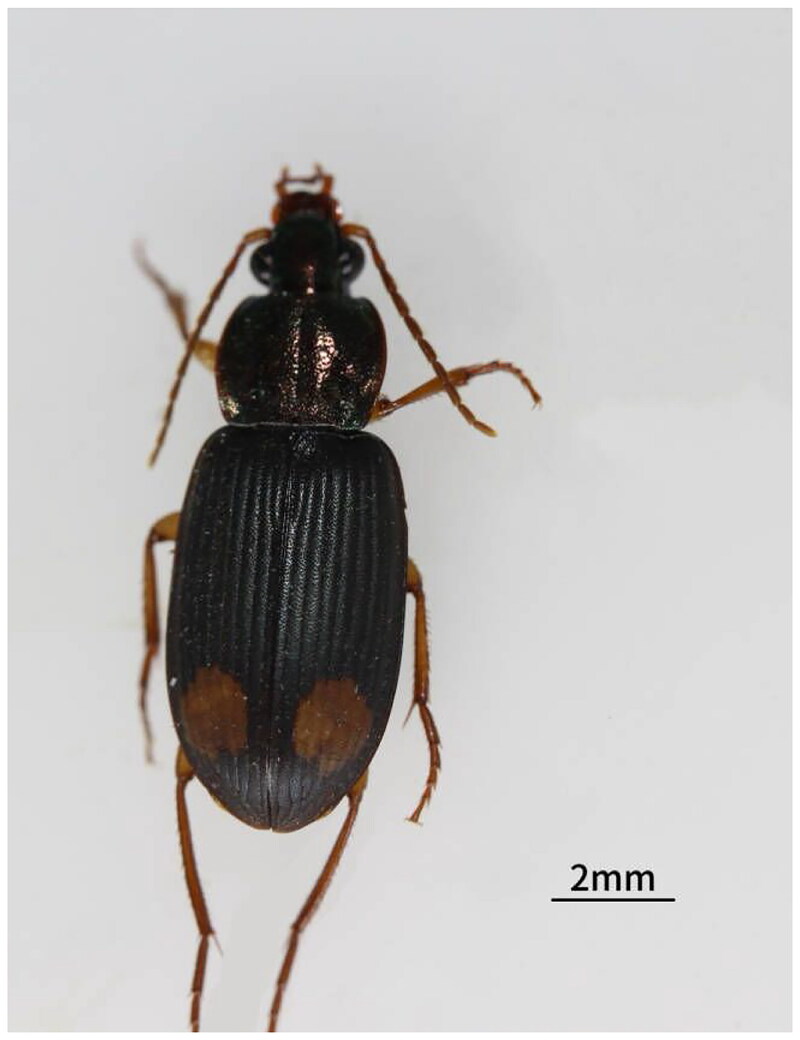
Morphological photograph of a female *Chlaenius bimaculatus* (photographed by Jun Li at Fujian Agriculture and Forestry University, Fuzhou City, China.).

*Chlaenius bimaculatus* Dejean, 1826 (Coleoptera: Carabidea) is distinguished by its golden cyan body, enlarged end segments of the lower lip and jaw whiskers, and a large reddish-yellow spot on the sub posterior portion of the forewing (Figure 1) (Li et al. 1990; Qiu 1996). *C. bimaculatus* overwinters as adults in weeds, humus-rich dirt clods, and stones before laying eggs in April the following year in Fujian Province, China, where it is often found in sweet potato and rice fields (Chen and Chen 1984).

Biological control is an essential strategy for the long-term control of *Spodoptera frugiperda* and other lepidopteran pests, with natural enemy insect resources being the most important (Chen et al. 2019). *C. bimaculatus* can effectively manage crop pests and reduce economic loses from agricultural products. *S. frugiperda*, *S. litura*, *Helcystogramma triannulella*, *Omiodes indicata*, and other lepidopteran pest larvae are preyed upon by both adults and larvae of *C. bimaculatus* (Chen and Chen 1982; 1991; Weng 1995; Huang et al. 2020). *C. bimaculatus* is a predatory natural predator with significant application potential, however, there has been little research on its utilization (Huang et al. 2022). Therefore, in this study, the complete mitochondrial genome sequence of *C. bimaculatus* was sequenced and annotated to provide data for future molecular studies on this species.

## 2. Materials

Adult *C. bimaculatus* specimens were collected from Lianjiang, Fujian Province, China (119°23′38″E, 26°3′7″N) using sexual attractant traps and subjected to complete mitochondrial genome sequencing for molecular phylogenetic study. The Key Laboratory of Integrated Pest Management in Ecological Forests, Fujian Agriculture and Forestry University (URL: https://lxy.fafu.edu.cn; contact person: Songqing Wu; email: dabinyang@126.com) served as the repository for voucher specimen BJ-202301.

## 3. Methods

### 3.1. Mitochondrial genome assembly and annotation

The leg of an adult *C. bimaculatus* individual was used for total genomic DNA extraction, which was subsequently purified using the TruSeq DNA Sample Preparation Kit (Vazyme, Fuzhou, China) and the QIAquick Gel Extraction Kit (Qiagen, Hilden, Germany). The quality and concentration of the extracted DNA were determined using a NanoDrop spectrophotometer (Thermo Fisher Scientific, Waltham, MA, USA). Then, DNA sequencing was performed on an Illumina HiSeq 2500 platform (Illumina, San Diego, CA, USA). The total sequencing data obtained for the mitotic genome was 6 Gb. After filtration steps were applied to remove low-quality reads and artifacts from the raw data set consisting of 61,332,980 reads in total; a final set of 3,835,348 clean reads remained. These clean reads were assembled using MitoZ (Meng et al. 2019) and metaSPAdes software (Nurk et al. 2017). The MITOs web server (Matthias et al. 2013) was used to annotate the assembly results based on the reference sequence of *Harpalus pensylvanicus* (GenBank accession number: NC_046953.1). The obtained genomic sequence data has been deposited in the NCBI database under accession number OR536810. Visualization of the mitogenome map was accomplished with Chloroplot software available at https://irscope.shinyapps.io/Chloroplot/. Additionally, transfer RNA (tRNA) genes were predicted utilizing tRNA scan software (Lowe & Eddy 1997).

### 3.2. Phylogenetic analysis

To gain a more comprehensive understanding of the taxonomic status of *C. bimaculatus*, we constructed a phylogenetic tree utilizing the complete mitochondrial genomes of *C. bimaculatus* and 15 other Carabidae species selected from NCBI-BLAST (http://blast.ncbi.nlm.nih.gov), with *Limonius californicus* (Coleoptera: Elateroidea; GenBank accession number: KT852377.1) serving as the outgroup (Table 1). The complete mitochondrial genomes were aligned using the MUSCLE alignment software (Edgar 2022). Subsequently, an evolutionary tree employing the maximum-likelihood (ML) method was generated in MEGA 7 with bootstrap values for 1000 replications (Kumar et al. 2016). Finally, iTol v6 (https://itol.embl.de/) was employed to visualize the resulting phylogenetic tree.

**Table 1.** Information on the mitogenomes of the species used in this study.

Superfamily	Family	Species	GenBank no.	References
Caraboidea	Carabidae	*Pterostichus madidus*	KT876910.1	Linard et al. (2016)
		*Pterostichus niger*	KT876909.1	Linard et al. (2016)
		*Stomis pumicatus*	KX087349.1	Unpublished
		*Amara communis*	NC_036268.1	Unpublished
		*Diplocheila zeelandica*	NC_067050.1	Unpublished
		*Chlaenius bimaculatus*	OR536810	This study
		*Harpalus griseus*	NC_066080.1	Unpublished
		*Harpalus anxius*	NC_066079.1	Unpublished
		*Blethisa multipunctata*	KX087243.1	Unpublished
		*Elaphrus cupreus*	KX087286.1	Unpublished
		*Carabus granulatus*	MN122850.1	Unpublished
		*Trachypachus holmbergi*	NC_011329.1	Unpublished
		*Brachinus crepitans*	JX412826.1	Ivanič Porhajašová and Babošová (2022)
		*Galerita orientalis*	NC_066084.1	Bai et al. (2022)
		*Harpalus sinicus*	NC_045094.1	Yu et al. (2019)
		*Harpalus pensylvanicus*	NC_046953.1	Kieran (2020)
Elateridae	Elateroidea	*Limonius californicus*	KT852377.1	Sandhi et al. (2022)

Note: These species used in this study are the complete mitochondrial genomes.

## 4. Results

**Figure 2.**
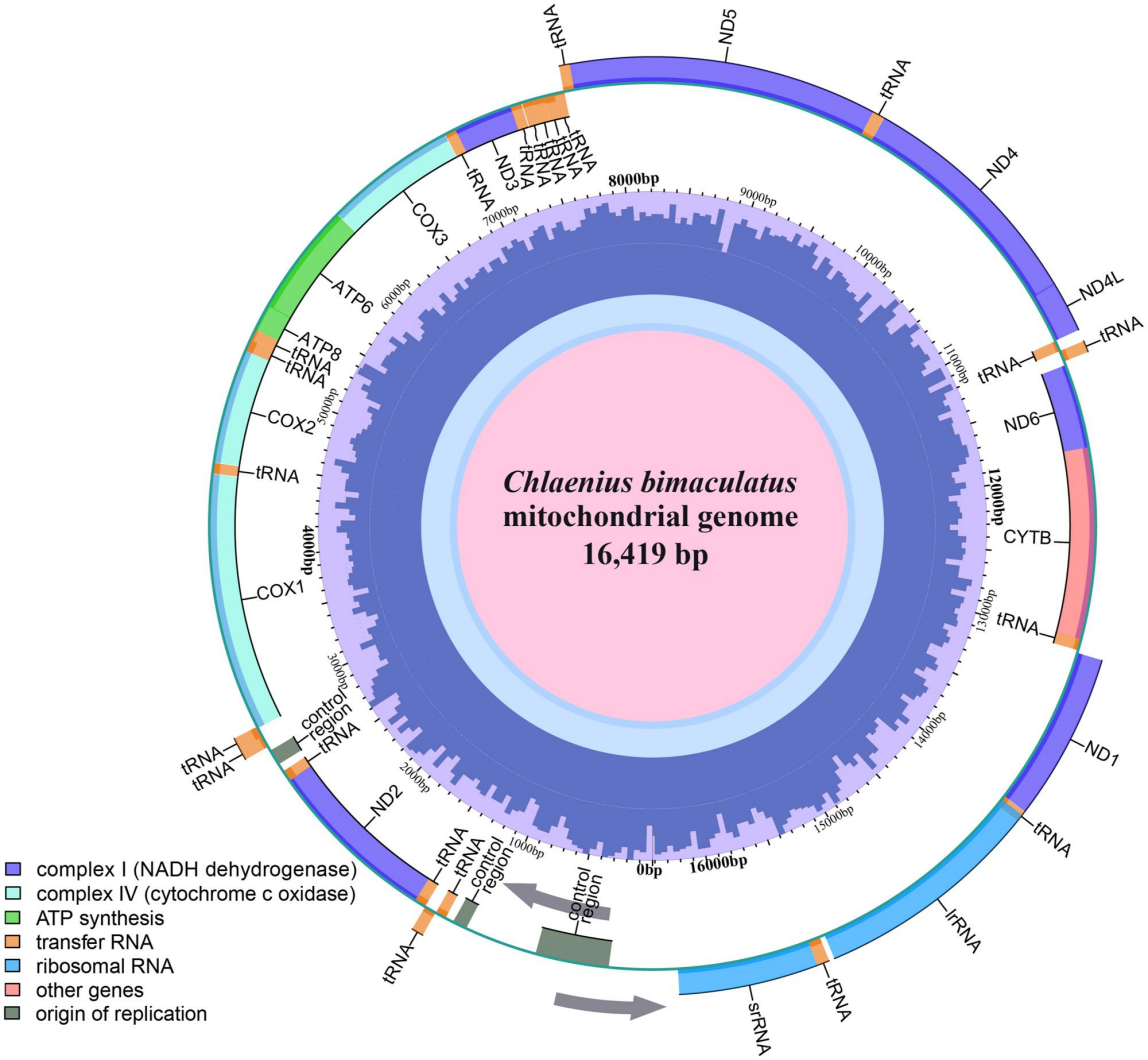
Mitogenome map of the mitochondrial genome of *Chlaenius bimaculatus* (GenBank: OR536810). The inner circle indicates the GC content, and the colors on the external circle indicate different genes and regions. The arrows represent the direction of transcription; genes encoded on the heavy and light strands are shown inside and outside the circle, respectively.

The mitogenome of *C. bimaculatus* was assembled based on the depth of the coverage (high coverage of over 5000×) (Figure S1). The complete mitochondrial genome of *C. bimaculatus* had a length of 16,419 bp and a GC content of 24% (*A* = 41%, *T* = 38.4%, *C* = 12%, *G* = 8.6%). It comprised 37 annotated genes, including 13 protein-coding genes (PCGs), two ribosomal RNA genes (tRNAs), and 22 transfer RNA genes (tRNAs). This genome arrangement is consistent with that commonly observed in other insects (Cameron 2014). Fourteen genes were transcribed on the minority strand (N-strand), consisting of eight tRNAs, four PCGs, and two rRNAs; while the remaining fourteen tRNAs and nine PCGs were transcribed on the majority strand (J-strand). The total length of the 13 PCGs was 11,085 bp, encoding a total of 3683 amino acids. The lengths of the 16S and 12S rRNA were measured as 1330 bp and 789 bp, respectively. Amongst the 22 tRNA molecules present in this genome assembly ranged from 60 bp (tRNA-Glu, tRNA-Phe) to 71 bp (tRNA-Val, tRNA-Lys) (Figure 2). Regarding start codons for protein-coding genes: COX1, ATP8, ND3, and ND6 utilized ATT as their start codon; COX2, ATP6, COX3, and CYTB employed ATG as their start codon; whereas ND5 and ND4L used TAA as their respective start codons. In contrast, the ND2 gene started with ATA, the ND1 gene began with AAC, and the ND4 gene initiated with TAC. Six genes (ND2, COX1, ATP8, ATP6, COX3, and ND6) terminated with TAA as their stop codon. ND3 and CYTB utilized TAG as a stop codon. ND4 and ND4L ended with ATT as a stop codon. The stop codons for COX2 and ND5 genes were completed by adding 3′ A residues to the mRNA.

**Figure 3.**
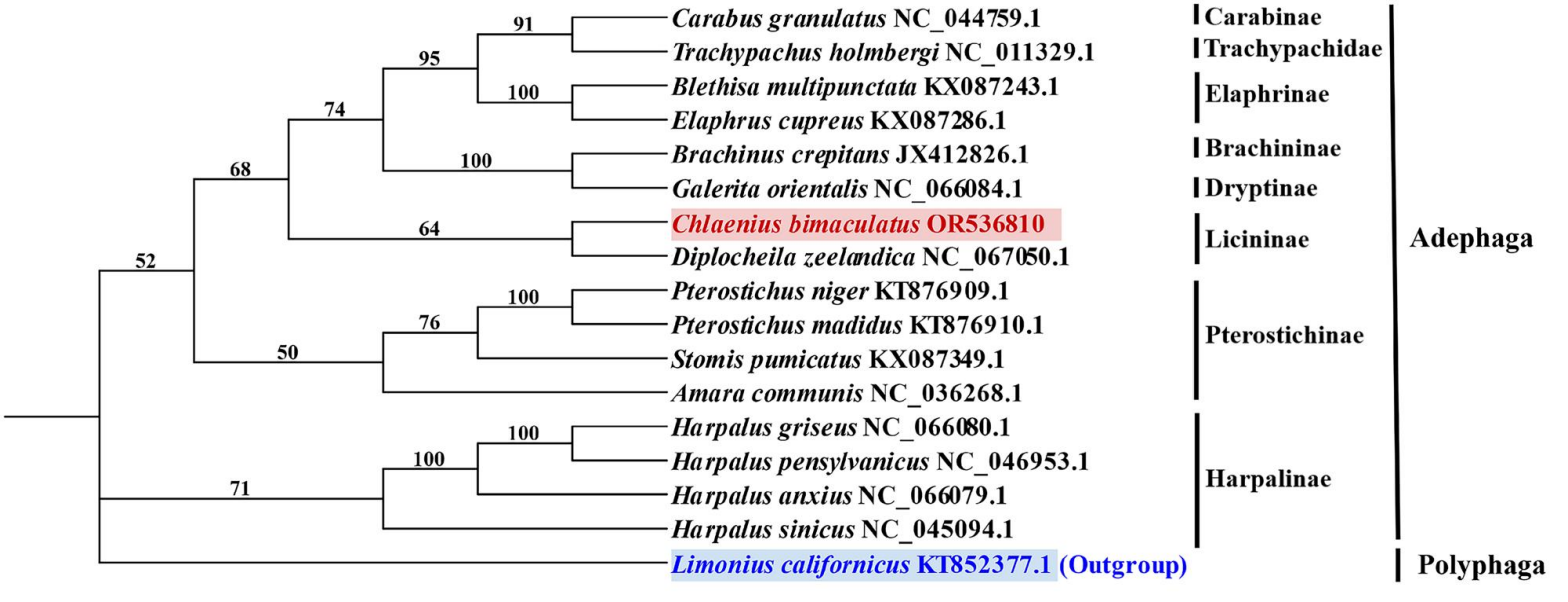
Maximum-likelihood analysis of *Chlaenius bimaculatus* and sixteen related species insects based on genome sequence with 1000 bootstraps. Bootstrap support values are labeled near the branch. The red text indicates which sequences were newly revealed in this study and the blue text indicates an outgroup.

The phylogenetic analysis revealed that the analyzed species were classified into three major clades. The first clade, located at the root of the tree, consisted of *L. californicus*, which belongs to the Polyphaga of Coleoptera. The remaining 16 species belonged to the Adephaga of Coleoptera. The second clade comprised *H. sinicus*, *H. anxius*, *H. pensylvanicus*, and *H. griseus* from the Harpalinae subfamily within Caraboidea. In addition, the third branch included *C. bimaculatus* and other species from the same family as *C. bimaculatus* was found to be closely related to *Diplocheila zeelandica* in terms of mitochondrial genomes; both *C. bimaculatus* and *D. zelandica* belonged to Licininae subfamily within Caraboidea (Figure 3).

## 5. Discussion and conclusions

In this study, we successfully sequenced and annotated the complete mitochondrial genome of *C. bimaculatus*. The total length of the mitochondrial gene group is 16,419 bp, with a GC content of 24%. Phylogenetic analysis based on the maximum-likelihood method revealed a close relationship between *C. bimaculatus* and the mitochondrial genome of *D. zeelandica*. This phylogenetic analysis provides crucial insights into the evolutionary dynamics of the mitochondrial genome in this taxonomic group, while also establishing an indispensable foundation for future genetic investigations. Moreover, the availability of comprehensive mitochondrial genomic data for *C. bimaculatus* will greatly facilitate advancements in developing novel biological control strategies against Lepidopteran pests such as *S. frugiperda*.

## Supplementary Material

Supplementary Material.docx

## Data Availability

The genome sequence data supporting this study’s findings are available in GenBank of NCBI at (https://www.ncbi.nlm.nih.gov) under assessment No. OR536810. The associated BioProject, Bio-Sample, and SRA numbers were PRJNA1024890, SAMN37704168, and SRS19092383.
