# Supplementary material for: The complete mitochondrial genome of Chlaenius bimaculatus Dejean, 1826 (Coleoptera: Carabidea) and its phylogenetic analyses
Source: Mitochondrial DNA B Resour. 2024 Sep 4;9(9):1170–4. doi: 10.1080/23802359.2024.2397993 (PMC11376285; doi:10.1080/23802359.2024.2397993)
Supplement: Supplementary Material.docx [file TMDN_A_2397993_SM2048.docx]

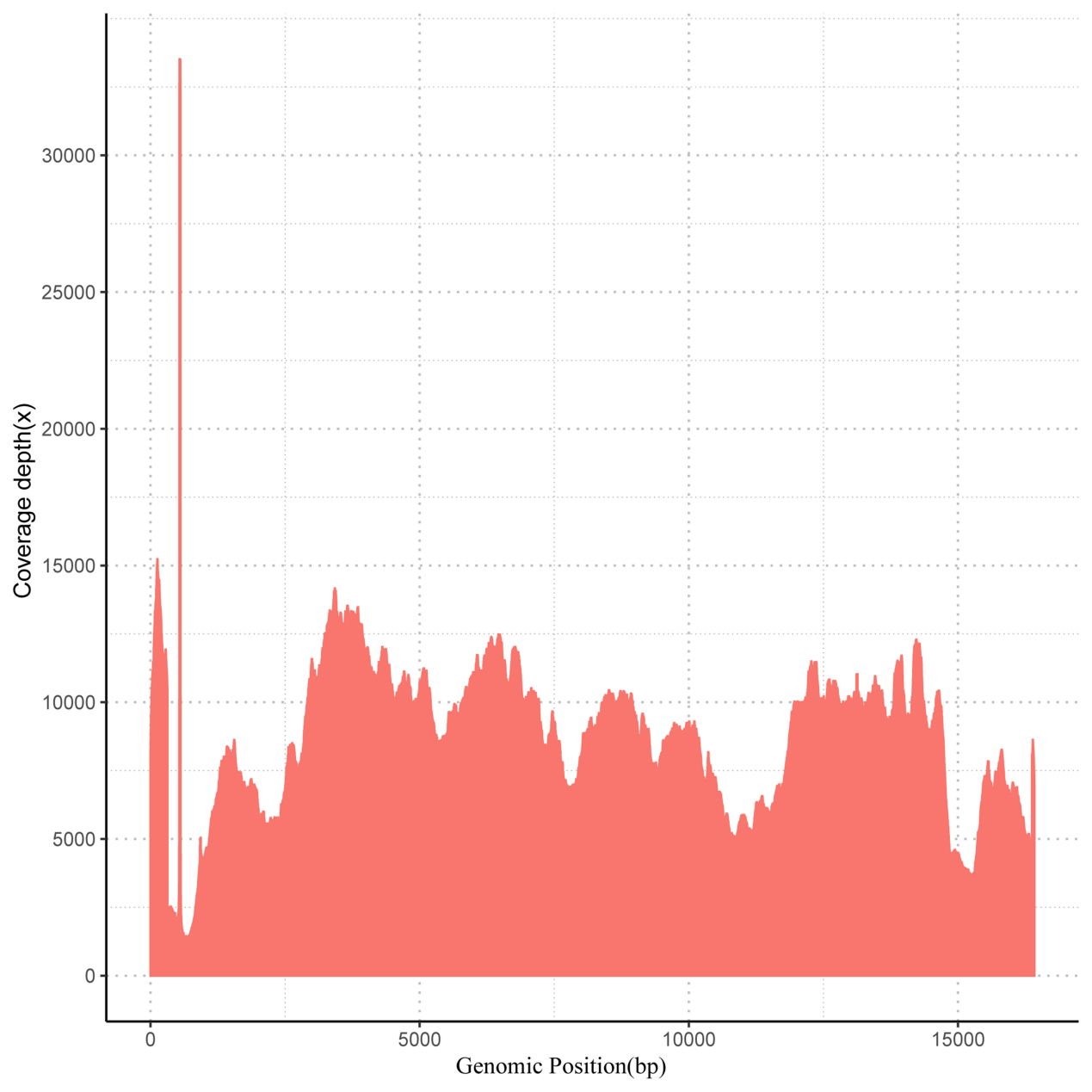


**Figure S1.** Depth of coverage for *Chlaenius bimaculatus* mitochondrial genome. X and Y axis present nucleotide position of *C. bimaculatus* mitochondrial genome and coverage depth, respectively.
